# Accuracy and dentin conservation of guided endodontic access versus conventional access in simulated calcified canals: A micro-CT *in vitro* analysis

**DOI:** 10.6026/973206300220689

**Published:** 2026-02-28

**Authors:** Shrutika Jadhav, Reethi Siri Sresty Taduvai, Gouri R Reddy, Sumaida, Suchismita Chakraborty, Mohammed Mustafa

**Affiliations:** 1 Department of Conservative Dentistry and Endodontics, DY Patil Dental School Lohegao Charoli Bk, Maharashtra, India; 2 Department of Dentistry, SDM College of Dental Sciences, Hubbali, Karnataka, India; 3 Department of Dentistry, Shri Atal Bihari Vajpayee Medical College and Research Institute, Bengaluru, India; 4 Department of Conservative Dentistry and Endodontics, Maharishi Markandeshwar College of Dental Sciences and Research, Mullana, Ambala, Haryana, India; 5 Department of Conservative Dentistry and Endodontics, Regional Dental College, Assam, India; 6 Department of Conservative Dental Sciences, College of Dentistry, Prince Sattam Bin Abdulaziz University, Al-Kharj 11942, Saudi Arabia

**Keywords:** Guided endodontics, pulp canal obliteration, micro-CT, dentin conservation, endodontic access

## Abstract

Pulp canal obliteration presents a significant challenge during conventional endodontic access, often resulting in procedural errors and excessive dentin removal. Therefore, it is of interest to evaluate the accuracy and dentin conservation of guided endodontic access compared with conventional freehand access in simulated calcified canals using micro-computed tomography. Forty extracted mandibular premolars with induced canal obliteration were randomly allocated to guided (n = 20) and conventional (n = 20) access groups and angular deviation, linear deviation at the apex, access cavity volume, remaining dentin thickness and procedure time were assessed. Guided access demonstrated significantly lower angular and linear deviations, reduced cavity volume and greater preservation of dentin thickness compared with the conventional approach (p < 0.001). Guided endodontic access is a more accurate and minimally invasive technique for managing calcified canals and may reduce iatrogenic complications while improving long-term treatment outcomes.

## Background:

Pulp canal obliteration is a pathological disease that consists of the gradual deposition of hard tissue into the root canal system to produce partial or full canal calcification [1]. It is common as a secondary effect of trauma of the teeth, chronic inflammatory events, aging, or orthodontics and prevalence rates of the condition have been reported to be between 4 and 24 percent after traumatic injuries [2]. There are significant problems in the clinical treatment of teeth with calcified canals as the reduced or lack of visualization of the canal considerably complicates the preparation of access cavity and the following negotiation of root canal [3]. Traditional endodontic access in the calcified canals depends on the level of tactile sensitivity, clinical experience as well as the anatomical knowledge of the operator to locate and navigate the obliterated canal space [4]. Such a freehand technique often causes procedural complications such as over-removal of dentin, perforation of the floor of the root or pulp chamber, loss of original canal direction and separation of instruments [5]. The research has shown that traditional access in calcified canals has a perforation rate of between 2.6 to 10.9 which is significantly higher than the non-calcified teeth [6]. Sound dentin preservation during the access cavity preparation is crucial to the structural integrity of endodontically treated teeth [7]. High incidence of dentin loss during access procedures dramatically implies fracture resistance and the studies have suggested that every millimetre of coronal dentin loss decreases the strength of the tooth by about 14 percent [8]. Increased loss of tooth structure has been linked to traditional endodontic access cavities, which has led to the emergence of minimally invasive endodontic concepts to maintain pericervical dentin [9]. The latest technological achievements have helped in the incorporation of the computer-aided design and manufacturing concepts in the practice of endodontics with the creation of guided endodontic access protocols [10]. This method involves the use of cone-beam computed tomography images with the help of digital surface scanning and special software to plan and make three-dimensional printed guides, which guide the access bur to follow a specific route to the target canal [11]. The guided method has the promise of such benefits as increased precision, shorter procedure duration, less dentin loss and fewer chances of iatrogenic mistakes [12]. A number of studies have compared the clinical and laboratory efficacy of guided endodontic procedures to give encouraging results [13]. Studies have established that guided access has angular and linear error rates of less than 2 and 0.5mm respectively at the target [14]. Moreover, guided methods have demonstrated excellent dentin preservation over traditional ones especially in problematic anatomy [15]. Micro-computed tomography is a new technology that is being used as the gold standard of 3D analysis of root canal anatomy and evaluation of endodontic procedures because of its high resolution and non-destructive capability [16]. This form of imaging allows a clear volumetric evaluation of the access cavities, measurement of the remaining thickness of the dentin, as well as the detailed examination of the results of the procedures [17]. Although there is an increasing amount of evidence to promote guided endodontic access, systematic comparative studies that use micro-CT analysis to investigate both the accuracy parameters and dentin conservation measures concurrently have not been done in large amounts [18]. Also, universalized guidelines to develop reproducible simulated calcification models to use in *in vitro* research must be developed further [19]. Therefore, it is of interest to determine the accuracy and conservation of dentin between guided access and conventional freehand access of micro-computed tomography analysis in simulated calcified canals. The hypothesis of the null was that there are no significant differences in guided and conventional access methods in terms of parameters of accuracy and dentin preservation.

## Materials and Methods:

## Design of the study and sample selection:

This experimental study was done *in vitro* and this was carried out after the institutional ethics committee gave consent. An initial calculation of the sample size was done using G + Power software (G + Power version 3.1.9.7) with an effect of 0.85, alpha error of 0.05 and power of 0.80 and would need at least 18 specimens per group. In a bid to take into consideration the possibility of specimen loss, 40 extracted human mandibular premolars were randomly chosen to include. Inclusion criteria were mandibular single- rooted premolars, full development of root, no caries or restorations, no cracks or fractures visible under magnification and a root length of 12-16mm. The exclusion criteria were teeth with more than one canal, root resorption, prior endodontic therapy, calcified canals and anatomic anomalies. The teeth were obtained at the age of 18-45 years after an extraction due to orthodontic or periodontal causes and informed consent was received to use the research.

## Canal obliteration simulation specimen preparation:

Ultrasonic scalers were used to clean off soft tissue debris and calculus of selected teeth and kept in 0.1% thymol solution at 4°C until used. The measurement of initial patency of all canals was done through inserting size 10 K-file into the apical foramen. The canal obliteration was modeled on a standardized protocol. The size 2 round diamond bur was used to prepare access cavities under water coolant. Rotary nickel-titanium files were used to instrument canals to a size 25/.06 and 2.5% sodium hypochlorite was used to irrigate the canals. Canals were then dried under paper points and obturated using mineral triite aggregate condensed to putty consistency under the cementoenamel junction up to 2mm below the cementoenamel junction. The specimens were kept in 100% humidity at 37°C after 72 hrs in order to settle fully. After obliteration, the access cavities were refilled with composite resin in an incremental approach with adhesive bonding, which mimicked the clinical appearance of teeth with a pulp canal obliteration that has already been restored or has intact coronal structure to cover the canals with calcification.

## Micro-CT scanning protocol:

Micro-CT scans were taken of pre-operative measuring with a high-resolution micro-CT scanner (SkyScan 1275, Bruker, Kontich, Belgium) with the following parameters: voltage 100kV, current 100MicroA, pixel size 10MicroM, rotation step 0.4, 360 rotation, aluminum/copper filter and frame averaging 3. The samples were placed in a tailor-made acrylic set to allow reproducible location in successive scans. The reconstruction was done with NRecon software (version 1.7.4.6, Bruker) with standard parameters such as beam hardening correction of 40, ring artifact reduction of 6 and smoothing of 1.

## Group allocation and treatment protocols group allocation and treatment protocols:

The samples were randomly assigned to two categories (n=20) of designs by computer-generated randomization:

[1] Group A - Guided Endodontic Access: Intraoral scanner (TRIOS 3, 3Shape, copenhagen, Denmark) obtained digital surface scans. Micro-CT DICOM data of the pre-operative micro-CT and surface scan STL files were loaded into an implant planning software (coDiagnostiX, Dental Wings, Montreal, Canada) altered to meet endodontic applications. The virtual planning considered the initial canal path to be in the MTA-filled canal area, the laying of a virtual drill path along the long axis of the canal and the design of a 1.3mm diameter drill sleeve to fit in the intended drill. The surgical guides were printed by stereolithography 3D printing (Form 3B, Form labs, Somerville, MA, USA) with surgical guide resin (Surgical Guide Resin, Form labs). The guides were washed with 99 percent isopropyl alcohol for 15 minutes and then dried at 60°C 30 minutes as recommended by the manufacturers. The procedure was done with the guide locked in place with a 1.0mm diameter trephine bur (CK Dental Specialties, Orange, CA, USA) at 800 rpm with light intermittent pressure and constant water irrigation. The bur was kept being drilled until it reached the depth that had been planned according to what the guide had drilled.

[2] Group B - Conventional Freehand Access: Access was done by an experienced endodontist and no digital guidance was used using the standard techniques. Initial access with the composite restoration was with a size 2 round diamond bur, then round tungsten carbide burs followed by ultrasonic tips (Start-X, Dentsply Sirona, Charlotte, NC, USA) under the magnification of the dental operating microscope (10-20x). Access was maintained until the calcified canal material was identified and penetrated as indicated by a tactile feeling and visualization of MTA debris.

## Post-operative assessment:

The micro-CT scans were taken in the same conditions and positioning of the specimen in post-operative stages. Co-registration of image datasets was done using data viewer software (version 1.5.6.2, Bruker) so that pre- and post-operative scans could be superimposed accurately.

## Outcome measurements:

## Accuracy parameters:

[1] Angular deviation (α): Angle between the planned/ideal path of access and the actual access path.

[2] Linear deviation at apices (mm): The difference between the planned target position and the actual final position at the apical end of the preparation of access.

[3] Linear deviation at coronal entrance (mm): The gap between the intended position of entry on the tooth surface and the real position of entry on the tooth surface.

## Parameters of dentin conservation:

[1] Access cavity volume (mm 3): The volume of removed tooth structure in total by use of CTAn software (version 1.20.3.0, Bruker).

[2] Thickness of remaining dentin (mm): The nearest distance between the access preparation and the external root surface, at the point of most criticality.

[3] Volumetric dentin loss (%): Percentage of initial dentin volume loss in preparing access.

## Procedural parameters:

[1] Procedural time (minutes): The time taken between the beginning of preparation of access and the established canal location.

[2] Perforation incidence: This is the iatrogenic perforation that has been confirmed through visual inspection and micro-CT analysis.

Measures were conducted on two blinded but group-allocation-blinded calibrated examiners. Intraclass correlation coefficient was used to measure inter-examiner reliability where a value above 0.90 is acceptable.

## Statistical analysis:

The SPSS software (version 27.0, IBM Corp., Armonk, NY, USA) was used in statistical analysis. Shapiro-Wilk tests were used to test the normality of the data distribution. Mean Standard deviation was used to represent continuous variables. The independent samples t-test was conducted where the sample data were normally distributed and the Mann-Whitney U test was used where the sample data was not normally distributed between the groups. The Fisher exact test was used to test categorical variables. The statistical level was determined to be at p>0.05.

## Results:

All 40 specimens completed the study protocol without exclusions. Inter-examiner reliability demonstrated excellent agreement with intraclass correlation coefficients ranging from 0.92 to 0.98 for all measured parameters. The guided endodontic access group demonstrated significantly superior accuracy across all measured parameters compared to the conventional access group. Mean angular deviation was 1.24±0.38°C in the guided group versus 4.87±1.52°C in the conventional group (p<0.001). Linear deviation at the apex was 0.31±0.12mm for guided access compared to 1.18±0.45mm for conventional access (p<0.001). Coronal entry point deviation was 0.18±0.07mm in the guided group versus 0.89±0.34mm in the conventional group (p<0.001). Detailed accuracy results are presented in Table 1.

## Dentin conservation parameters:

Guided endodontic access resulted in significantly better dentin preservation compared to conventional access. Mean access cavity volume was 12.45±2.31mm^3^ in the guided group compared to 18.72±3.84mm^3^ in the conventional group (p<0.001). Minimum remaining dentin thickness was 1.42±0.18mm for guided access versus 0.98±0.24mm for conventional access (p<0.001). Volumetric dentin loss was 8.34±1.56% in the guided group compared to 12.87±2.45% in the conventional group (p<0.001). Complete dentin conservation results are shown in Table 2.

## Procedural parameters and complications:

Mean procedural time was significantly longer in the conventional access group (24.35±8.42 minutes) compared to the guided access group (8.67±2.14 minutes, p<0.001), excluding guide design and fabrication time. No perforations occurred in the guided access group, while 3 perforations (15%) were observed in the conventional group (p=0.231, Fisher's exact test). All perforations in the conventional group were lateral root perforations occurring in the middle third of the root. Procedural outcomes are summarized in Table 3. The success rate for canal location was 100% in the guided group compared to 85% in the conventional group. Three specimens in the conventional group experienced either perforation (n=3) resulting in failed canal location; however, this difference did not reach statistical significance (p=0.231).

## Discussion:

The results of the research conclude that guided endodontic access has far better accuracy and better dentin preservation than the traditional freehand access in simulated calcified canals. The findings confirm the null hypothesis rejection and concur with the expanding literature on the support of computer-guided methods in problematic endodontic cases [20]. The angular deviation of the guided access group (1.241/38) is also in the same range of the clinically acceptable range of previous studies, which have reported an angular deviation of 0.9 to 2.8 in between guided techniques [21]. This accuracy is explained by the strict control of the 3D-printed template that limits the access bur to the fixed path and reduces the effect of the operator deviation [22]. The traditional access group, in contrast, had significantly greater angular deviation (4.87±1.52) owing to the natural difficulty of having to navigate through obliterated canal systems using only free hand navigation, in which there are no visual or tactile cues to guide you [23]. The linear deviation at the apex is a paramount accuracy characteristic because deviations surpassing anatomical intolerances can lead to miss canals, ledge or perforation [24]. The guided group demonstrated mean apical linear deviation of 0.31+0.12mm, which is in agreement with systematic reviews that show pooled mean deviation of 0.17mm to 0.47mm with endodontic guided techniques [25]. The significantly increased deviation of the conventional group (1.18±0.45mm) underlines the technical challenge of keeping the drill to the correct path when long drilling through calcified tissue without guiding it [26]. One of the major benefits of the guided technique was dentin conservation. The volume of the access cavity in guided group (12.45+2.31mm 3) was nearly 33% lower than conventional group (18.72+3.84mm 3). Such loss of removed tooth structure has significant clinical implications which conservation of both coronal and radicular dentin has been directly related to increased fracture resistance of endodontically treated teeth [27]. Recent finite element simulation has shown that access preparations that are designed to be minimally invasive are a better distribution of functional stresses than access designs that were adopted in the past [28]. The least thickness of the dentin is a critical parameter to evaluate the risk of root perforation and structural integrity in the long run [29]. The guided group had much more remaining dentin thickness (1.42 0.18mm) than the conventional one (0.98 0.24mm). Studies have proposed that a dent in thickness less than 1mm is a significant risk of perforation in the next procedures and resistance against the pull of lateral forces [30]. It is worth noting that 35% of the traditional access preparations led to the presence of the remaining dentin thickness of less than 1mm in thickness, as opposed to 0 in the guided group. The time course of the procedures showed that guided access took much less chairside time (8.67±2.14 minutes) than traditional access (24.35±8.42 minutes). This analogy however does not include the amount of time it took to design and fabricate a guide which usually involves an additional 30-60 minutes to the workflow based on the level of software knowledge and the ability to manufacture such a guide [31]. The modern advancements in automated segmentation algorithms and simplified digital processes still decrease this extra time load [32]. The rate of perforation found in the conventional access group of 15% which, although not found to be statistically significant because of the sample size limitation, is a clinically alarming rate of complication common with other reports of iatrogenic errors during access to calcified teeth in the past [33]. The lack of perforations in the guided group is an indicator of the protective ability of predetermined planning of trajectories that consider root anatomy and danger zones [34].

The current results should be viewed in the framework of a number of limitations. *in vitro* nature of the study removes the clinical variables such as restricted mouth-opening, patient movements, hemorrhage and visibility limitations that could affect the real-life results [35]. Although the simulated calcification model with MTA offers uniform conditions in the comparison, it might not be ideal to simulate the different density and distribution trends of natural pulp canal obliteration [36]. Moreover, the experiment used a single experienced operator and results can be different depending on the familiarity of the operators with guided and standard methods [37]. Guided access of the endodontic procedure is cost-effective and must be considered in clinical implementation decisions. The strategy will require the investment in planning software, 3D printing technology and special drilling components [38]. Nevertheless, comparing it to possible expenditures on the treatment of procedural complications, e.g. perforations or root fracture, the guided method can prove positive cost-benefit properties, especially in high-risk cases [39]. The research directions to be pursued in the future are the randomized clinical trials of the patient-centered outcomes and then the success rate of the teeth treated by guided and conventional access methods in the long run [40]. Also, automated canal recognition and planning of treatment through the implementation of artificial intelligence is another area that can be further optimized when used to improve guided endodontic systems [41].

## Conclusion:

Guided endodontic access demonstrated significantly greater accuracy than conventional freehand access in simulated calcified canals, with markedly reduced angular and linear deviations. The guided approach preserved more dentin, required smaller access cavity volumes, reduced procedure time and minimized the risk of perforation compared with traditional methods. These findings support the clinical applicability of guided endodontic access as a predictable and conservative technique for managing pulp canal obliteration while preserving tooth structure.

## Figures and Tables

**Table 1:** Accuracy parameters comparison between groups

**Parameter**	**Guided Access (n=20)**	**Conventional Access (n=20)**	**p-value**
Angular deviation (°C)	1.24±0.38	4.87±1.52	<0.001*
Linear deviation at apex (mm)	0.31±0.12	1.18±0.45	<0.001*
Linear deviation at coronal entry (mm)	0.18±0.07	0.89±0.34	<0.001*
Maximum deviation along path (mm)	0.42±0.15	1.34±0.51	<0.001*
Deviation from canal long axis (°C)	0.98±0.31	3.92±1.28	<0.001*
*Values presented as mean ±
standard deviation;
Statistically significant (p<0.05)

**Table 2:** Dentin conservation parameters comparison between groups

**Parameter**	**Guided Access (n=20)**	**Conventional Access (n=20)**	**p-value**
Access cavity volume (mm^3^)	12.45±2.31	18.72±3.84	<0.001*
Minimum remaining dentin thickness (mm)	1.42±0.18	0.98±0.24	<0.001*
Mean remaining dentin thickness (mm)	1.89±0.22	1.45±0.31	<0.001*
Volumetric dentin loss (%)	8.34±1.56	12.87±2.45	<0.001*
Cervical dentin removed (mm^3^)	4.21±0.89	7.56±1.67	<0.001*
*Values presented as mean ±
standard deviation;
Statistically significant (p<0.05)

**Table 3:** Procedural parameters and complications

**Parameter**	**Guided Access (n=20)**	**Conventional Access (n=20)**	**p-value**
Procedural time (minutes)	8.67±2.14	24.35±8.42	<0.001*
Perforation incidence, n (%)	0 (0%)	3 (15%)	0.231
Canal location success, n (%)	20 (100%)	17 (85%)	0.231
Number of bur changes	0.15±0.37	2.45±0.89	<0.001*
Microscope adjustment frequency	0.20±0.41	4.85±1.73	<0.001*
*Values presented as mean ±
standard deviation or n (%);
Statistically significant (p<0.05)
